# Supplementary material for: Identification of photoperiod-induced specific miRNAs in the adrenal glands of Sunite sheep (Ovis aries)
Source: Front Vet Sci. 2022 Jul 22;9:888207. doi: 10.3389/fvets.2022.888207 (PMC9354845; doi:10.3389/fvets.2022.888207)
Supplement: Supplementary file 1 [file Data_Sheet_1.ZIP › Supplementary file/miRDeep2 file.zip!/miRDeep2 file/Know_Structure/LP42A3/oar-mir-125b.pdf]

miRBase precursor : oar-mir-125b
Total read count : 207279
oar-miR-125b read count : 206512
remaining reads : 767

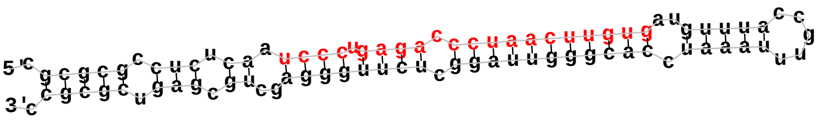

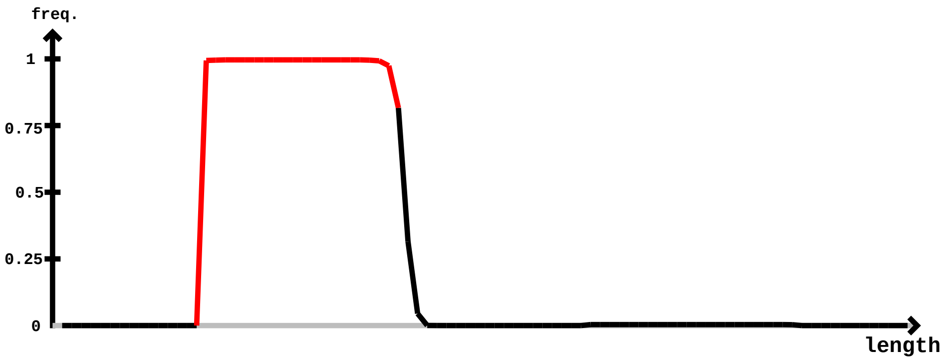


```
                 oar-miR-125b
5'- cgcgcgccucucaaucccugagacccuaacuugugauguuuaccguuuaaauccacggguuaggcucuugggagcugcgagucgcgcc   -3'    exp
    .(((((.(((.((.((((.((((.(((((((((((..(((((.....))))).)))))))))))).))))))))..)).))).))))).    reads      mm          sample
    .............Gucccugagacccuaacuugug.....................................................    1          1           seq
    .............aucccugagacccuaacuuguga....................................................    1          0           seq
    .............aucccugagacccuaacuugugau...................................................    1          0           seq
    ..............ucccugagacccuaacC.........................................................    29         1           seq
    ..............ucccugagaUccuaacu.........................................................    1          1           seq
    ..............ucccugagGcccuaacu.........................................................    1          1           seq
    ..............ucccAgagacccuaacu.........................................................    1          1           seq
    ..............Acccugagacccuaacu.........................................................    2          1           seq
    ..............ucccugagacccuGacu.........................................................    1          1           seq
    ..............ucccugagacccuaacu.........................................................    196        0           seq
    ..............uUccugagacccuaacu.........................................................    1          1           seq
    ..............ucccugagacccuaacCu........................................................    5          1           seq
    ..............ucccugagacccuaacuu........................................................    529        0           seq
    ..............ucccuAagacccuaacuu........................................................    1          1           seq
    ..............ucccugaCacccuaacuug.......................................................    1          1           seq
    ..............ucccugagacccuaacuug.......................................................    3819       0           seq
    ..............ucccugagacccuaacuugu......................................................    32758      0           seq
    ..............ucccugagacccuaacuugug.....................................................    103789     0           seq
    ..............ucccugagacccuaacuuguga....................................................    55646      0           seq
    ..............ucccugagacccuaacuugugau...................................................    6622       0           seq
    ..............ucccugagacccuaacuugugaG...................................................    2579       1           seq
    ..............ucccugagacccuaacuugugaGg..................................................    22         1           seq
    ..............ucccugagacccuaacuugugaug..................................................    34         0           seq
    ..............ucccugagacccuaacuugugaugu.................................................    21         0           seq
    ...............cccugagacccuaacuug.......................................................    3          0           seq
    ...............cccugagacccuaacuugu......................................................    11         0           seq
    ...............cccugagacccuaacuugug.....................................................    23         0           seq
    ...............cccugagacccuaacuuguga....................................................    195        0           seq
    ...............cccugagacccuaacuugugaG...................................................    8          1           seq
    ...............cccugagacccuaacuugugau...................................................    9          0           seq
    ................ccugagacccuaacuug.......................................................    1          0           seq
    ................ccugagacccuaacuugu......................................................    36         0           seq
    ................ccugagacccuaacuugug.....................................................    97         0           seq
    ................ccugagacccuaacuuguga....................................................    54         0           seq
    ................ccugagacccuaacuugugaG...................................................    2          1           seq
```

oar-miR-125b

```
cgcgcgccucucaaucccugagacccuaacuugugauguuuaccguuuaaauccacggguuaggcucuugggagcugcgagucgcgcc
................ccugagacccuaacuugugau...................................................    9     0     seq
.................cugagacccuaacuugug.....................................................    1     0     seq
.................cugagacccuaacuuguga....................................................    1     0     seq
..................ugagacccuaacuugug.....................................................    1     0     seq
.....................................................cacggguuaggcucuuggga...............    1     0     seq
.....................................................cacggguuaggcucuugggagc.............    2     0     seq
.....................................................cacggguuaggcucuugggagcug...........    1     0     seq
......................................................acggguuaggcucuuggg................    2     0     seq
......................................................acggguuaggcucuuggga...............    2     0     seq
......................................................acggguuaggcucuugggag..............    4     0     seq
......................................................acggguuaggcucuugggagc.............    86    0     seq
......................................................acggguuaggcucuugggagcu............    663   0     seq
......................................................acggguuaggcucuugggagcug...........    6     0     seq
```
